# Enhanced production of chitin deacetylase by *Penicillium oxalicum* SAE_M_-51 through response surface optimization of fermentation conditions

**DOI:** 10.1007/s13205-013-0118-4

**Published:** 2013-02-05

**Authors:** Nidhi Pareek, Sanjoy Ghosh, R. P. Singh, V. Vivekanand

**Affiliations:** 1 Department of Biotechnology, Indian Institute of Technology Roorkee, Roorkee, 247667 Uttarakhand India; 2 Present Address: Department of Chemistry, Umeå University, 90183 Umeå, Sweden; 3 Present Address: Protein Engineering and Proteomics Group, Department of Chemistry, Biotechnology and Food Sciences, Norwegian University of Life Sciences, 1430 Ås, Norway

**Keywords:** Central composite design, *Penicillium oxalicum*, Fermentation conditions, Chitin deacetylase, Chitosan

## Abstract

Optimization of the fermentation conditions for chitin deacetylase (CDA) production by *Penicillium oxalicum* SAE_M_-51 was undertaken in the present study using central composite design (CCD) under submerged condition. CDA is widely employed for bio-catalytic conversion of chitin to chitosan. Chitosan is a biopolymer with immense commercial potential in diverse industrial sectors, viz. pharmaceutics, food, agriculture, water treatment, etc. CDA production was significantly affected by all the variables studied, viz. pH, temperature, inoculum age and size. The optimal conditions that stimulating maximal CDA production were found to be: pH, 7.9; temperature, 28 °C; inoculum age, 90 h, and 11 % inoculum size. Under these optimized conditions, the actual maximal CDA production was 623.57 ± 8.2 Ul^−1^, which was in good agreement with the values predicted by the quadratic model (648.24 Ul^−1^), confirming the validity of the model. Optimization of fermentation conditions through CCD had resulted into 1.4-fold enhancement in CDA productivity (*Q*_p_ = 4.3264 Ul^−1^ h^−1^). Results of these experiments indicated that response surface methodology was proved to be a promising method for optimization of CDA production.

## Introduction

Modern era is the era of biotechnology for the production and application of various bio-based products. Biopolymers viz. cellulose, xylan, lignin, chitin, etc. are achieving prominence in a number of industrial sectors. Among all the polymers, recently commercial potential of chitin and its soluble derivatives has been explored a lot (Synowiecki and Al-Khateeb 2003; Dutta et al. 2004; Aranaz et al. 2009; Khoushab and Yamabhai 2010). Chitosan, the *N*-deacetylated derivative of chitin has enormous commercial potential due to its competitive properties like biodegradability, biocompatibility, solubility, non-toxicity, etc. (Kurita 2006). Though, chitosan is present in small amounts as animal biomass in the shells or cuticles of many crustaceans and also in the fungal cell wall, mostly it is derived from chitin by chemical or bio-catalytic alkaline deacetylation process. Chemical process needs a large amount of concentrated alkali, which in turn causes environmental concern. Also alkaline deacetylation process leads to the degradation of product quality with generation of heterogeneous products (Chang et al. 1997). Bioconversion is a competent alternative to the chemical one as it enjoys many merits over chemical process. Chitin deacetylase (CDA, EC 3.5.1.41) catalyzes the deacetylation of monomeric acetyl glucosamine residues of chitin by multiple attack mechanism and produces high quality chitosan (Tsigos et al. 2000). In the past few years, numerous microorganisms with CDA activity have been explored but most of them are intracellular CDA producers with limited yield (Araki and Ito 1975; Tsigos and Bouriotis 1995; Nahar et al. 2004; Cai et al. 2006; Kadokura et al. 2007).

For industrial application, exploration of novel CDA hyper producers with further optimization of production process is of utmost importance. But most of the research carried out so far has been related to the purification and characterization of CDA. Optimization of processing parameters plays an important role in the development of any fermentation process owing to their impact on the economy and efficacy of the process. The conventional approach of optimizing one-factor-at-a-time is laborious, time-consuming, and cannot provide the information on mutual interactions of the variables on the desired outcome. On the other hand, statistical experimental designs provide a systematic and efficient plan for experimentation to achieve certain goals so that many factors can be simultaneously studied (Bas and Boyaci 2007). Statistical data analysis allows visualization of the interactions among several experimental variables by carrying out a limited number of experiments, leading to the prediction of data in the areas not directly covered by experimentation. Response surface methodology (RSM) is proved to be an important tool to study the effect of both the primary factors and their mutual interactions on the response to determine the optimal conditions. The statistical programs create several classes of response surface designs. Among these, central composite design (CCD) is the most popular as it is very much flexible and allows sequential run of large number of experiments (Montgomery 2000).

Central composite design was carried out in the present investigation to optimize the fermentation parameters to enhance CDA production by mutant *P. oxalicum* SAE_M_-51 under submerged fermentation. To the best of our knowledge, this is the first report on the optimization of culture conditions for the CDA production by a heterotrophic fungus under submerged condition.

## Materials and methods

### Microorganism

*Penicillium oxalicum* SAE_M_-51 developed in our laboratory from the wild type *P. oxalicum* ITCC 6965 isolated from residual materials from sea food processing industry (Saubhagya Seafoods, Porbandar Gujarat, India) after successive stages of mutagenesis using microwave irradiation and ethidium bromide (EtBr) (Pareek et al. 2011a) was used in this study. The strain was maintained on potato-dextrose-agar (PDA) slants and stored at 4 °C.

### Enzyme production

For enzyme production, 50 ml of media in 250 ml Erlenmeyer flask was inoculated with *Penicillium oxalicum* SAE_M_-51 and incubated (30 °C, 6 days, 200 rpm). The fermentation medium contained (g l^−1^): Glucose, 10; yeast extract, 3.165; peptone, 4.754; KH_2_PO_4_, 3.0; K_2_HPO_4_, 1.0; MgSO_4_, 0.535; (NH_4_)_2_SO_4_, 0.255; NaCl, 0.5 and CaCl_2_, 0.5 and pH was adjusted to 6.0 (Pareek et al. 2011b). Following incubation, fermentation broth was centrifuged (8000*g*, 10 min) and supernatant was used for enzyme assay.

### CDA assay

Chitin deacetylase was assayed using ethylene glycol chitin as a substrate according to the method of Kauss and Bausch (1988). One unit of enzyme was defined as the activity which released 1 μmol of acetate from ethylene glycol chitin per minute under assay conditions.

### Optimization by response surface methodology

Central composite design was employed in the present investigation to optimize the fermentation conditions, viz. pH, temperature, inoculum age, and inoculum size for enhanced CDA production. It has a few steps viz. initial determination of the optimum region for the variables, behavior of the response in the optimum region, estimation of the optimal condition and verification (Khuri and Cornell 1987; Myers and Montgomery 1995). Each factor in the design was studied at five levels (−α, −1, 0, +1, +α, where α = 2^*k*/4^, *k* is the number of variables) (Table 1) in a set of 30 experiments that including 8 axial points, 16 factorial points, and 6 center points (Table 2). All experiments were conducted in triplicates and the mean value of CDA activity (Ul^−1^) was taken as the response (*Y*). A second-order polynomial equation with interaction terms was then fitted to the data by multiple regression analyses. This resulted in an empirical model that related the response measured to the independent variables of the experiment. For a four factor system the second order polynomial equation is (Eq. 1),

**Table 1:** Concentration ranges of four independent variables used in central composite design

Variable	Component	Range studied	Levels				
			−α	−1	0	+1	+α
*X*_1_	pH	4.00–8.00	4.00	5.00	6.00	7.00	8.00
*X*_2_	Temperature (°C)	20.00–40.00	20.00	25.00	30.00	35.00	40.00
*X*_3_	Inoculum age (h)	72.00–196.00	72.00	96.00	120.00	144.00	168.00
*X*_4_	Inoculum size (%, v/v)	1.00–13.00	1.00	4.00	7.00	10.00	13.00

α = 2^*k*/4^, where *k* = 4 represents no. of variables

**Table 2:** Experimental design matrix for optimization of chitin deacetylase production using central composite design

Run	Independent variables (actual values)				CDA (Ul^−1^)	
	pH	Temperature (°C)	Inoculum age (h)	Inoculum size (%)	Observed	Predicted
1	5.0	25.0	96.0	4.0	527.85 ± 3.2	520.75
2	5.0	25.0	96.0	10.0	543.16 ± 4.1	545.17
3	5.0	25.0	144.0	4.0	475.32 ± 2.8	478.63
4	5.0	25.0	144.0	10.0	528.73 ± 2.5	523.42
5	5.0	35.0	96.0	4.0	559.26 ± 3.4	550.96
6	5.0	35.0	96.0	10.0	558.14 ± 2.1	552.75
7	5.0	35.0	144.0	4.0	490.25 ± 3.1	491.08
8	5.0	35.0	144.0	10.0	516.35 ± 2.7	513.13
9	7.0	25.0	96.0	4.0	539.03 ± 1.8	545.96
10	7.0	25.0	96.0	10.0	568.34 ± 2.3	568.75
11	7.0	25.0	144.0	4.0	513.23 ± 3.4	520.08
12	7.0	25.0	144.0	10.0	551.76 ± 2.9	563.13
13	7.0	35.0	96.0	4.0	581.28 ± 3.3	587.42
14	7.0	35.0	96.0	10.0	587.87 ± 4.1	587.46
15	7.0	35.0	144.0	4.0	542.84 ± 3.8	545.17
16	7.0	35.0	144.0	10.0	556.54 ± 3.4	564.08
17	8.0	30.0	120.0	7.0	629.74 ± 2.7	610.13
18	4.0	30.0	120.0	7.0	521.71 ± 2.5	533.96
19	6.0	40.0	120.0	7.0	540.65 ± 2.4	542.13
20	6.0	20.0	120.0	7.0	519.85 ± 3.2	510.96
21	6.0	30.0	168.0	7.0	472.16 ± 1.7	461.79
22	6.0	30.0	72.0	7.0	523.37 ± 2.4	527.29
23	6.0	30.0	120.0	13.0	596.39 ± 3.5	593.46
24	6.0	30.0	120.0	1.0	552.75 ± 2.6	548.63
25	6.0	30.0	120.0	7.0	546.24 ± 2.3	545.17
26	6.0	30.0	120.0	7.0	542.08 ± 3.1	545.17
27	6.0	30.0	120.0	7.0	543.61 ± 1.6	545.17
28	6.0	30.0	120.0	7.0	545.29 ± 3.5	545.17
29	6.0	30.0	120.0	7.0	547.66 ± 2.4	545.17
30	6.0	30.0	120.0	7.0	548.15 ± 2.6	545.17

1where *Y* is the predicted response, *β*_0_ is offset term, *β*_*i*_ are coefficients for the linear terms, *β*_*ii*_ are for the square terms, *β*_*ij*_ are the coefficients of interactive terms.

The statistical software package ‘Design-Expert 8.0’, StatEase, Inc., Minneapolis, USA was used to analyze the experimental design. The optimum levels of pH, temperature, inoculum age, and inoculum size, to achieve maximum CDA production were obtained by solving the regression equation and also by analyzing the response surface contour plots.

### Validation of the experimental model

Validation of the model and regression equation was performed under the conditions predicted by the model. Samples were drawn at desired intervals and analyzed for CDA production as described earlier.

## Results and discussion

Present investigation was carried out to optimize fermentation conditions to achieve maximal CDA production under submerged condition for bioconversion of chitin to chitosan.

### Regression analysis

The effect of four culture variables (pH, temperature, inoculum age and inoculum size) on CDA production was determined by CCD. The mean predicted and observed responses are given in Table 2. The results obtained by CCD were analyzed by analysis of variance (ANOVA) (Table 3). By applying multiple regression analyses on the experimental data, the following second order polynomial equation was found to explain the CDA production as a function of the four variables studied (Eq. 2):

**Table 3:** ANOVA for quadratic model

Source	SS	DF	MS	*F* value	*P* value (Prob > *F*)
Model	29,794.55	14	2,128.18	24.61	<0.0001
Residual	1,296.92	15	86.46	–	–
Lack of fit	1,270.08	10	127.01	23.67	0.0014
Pure error	26.83	5	5.37	–	–
Total	31,091.47	29	–	–	–

*R*^*2*^: 0.9583; Adj *R*^*2*^: 0.9194; C.V.: 1.72 %; Adeq. precision: 22.560

2where *Y* represents CDA production (Ul^−1^) and *X*_1_, *X*_2_, *X*_3_, and *X*_4_ are coded values of pH, temperature, inoculum age, and inoculum size, respectively.

The Fisher’s *F* test for the model with a very low probability value [(*P*_model_ > *F*) < 0.0001] indicates the significance of the model. ANOVA for CDA production indicated the ‘*F* value’ to be 24.61, which implied that the model is highly significant in approximating the response surface of the experimental design. Corresponding probability of failure value (>*F*), tells that only 0.01 % chances are there that this value could occur due to noise. The goodness of fit of the model was examined by coefficient of determination (*R*^2^). Coefficient of determination (*R*^2^) for CDA production was calculated to be 0.9583, which can explain up to 95.83 % variability of the response. Adequate precision is a measure of the signal to noise ratio and a value greater than four is generally desirable. The ‘adequate precision’ value of 22.56 indicated an adequate signal and suggested that the model can be used to navigate the design space. The low value of coefficient of variance (CV = 1.72) indicated that experiments are conducted with very high precision. The “lack of fit test” compares the residual error to the pure error from replicated design points. The *F* value (23.67) for lack of fit test implies that it is highly significant and there is only 0.14 % chance that this large value could occur due to noise. The significance of each coefficient on the response was determined by Student’s *t* test and Probability of failure value and is given in Table 4. Model terms having values of ‘Prob > *F*’ less than 0.05 were considered significant, whereas those greater than 0.10 are insignificant. According to the present model, CDA production is significantly affected by the linear and squared terms of pH, temperature, inoculum age, and inoculum size. In addition, interactions of inoculum size with temperature and inoculum age were also observed to have significant effect on CDA production (*P* value <0.05). Effect of interaction between temperature and inoculum age (*P* value <0.10) can also be considered as noteworthy for CDA production. However, other interactions, viz. pH and temperature, pH and inoculum age, pH and inoculum size, were observed to be insignificant.

**Table 4:** Model coefficient estimated by regression analyses

Variables	*F* value	*P* value
Intercept	–	–
*X*_1_	100.65	<0.0001
*X*_2_	16.85	0.0009
*X*_3_	74.43	<0.0001
*X*_4_	34.87	<0.0001
*X*_1_^2^	14.32	0.0018
*X*_2_^2^	6.88	0.0192
*X*_3_^2^	50.82	<0.0001
*X*_4_^2^	13.27	0.0024
*X*_1_ *X*_2_	1.46	0.2450
*X*_1_ *X*_3_	3.05	0.1010
*X*_1_ *X*_4_	0.035	0.8532
*X*_2_ *X*_3_	3.64	0.0756
*X*_2_ *X*_4_	5.99	0.0272
*X*_3_ *X*_4_	4.74	0.0458

### Determination of optimum conditions

The advantage of utilizing RSM for optimization studies is to gain an idea about the effect of interaction of independent variables on the response. Derivation of the optimum level of each variable was performed by plotting response surface contour plots against any two independent variables, while keeping other two variables at their respective zero levels.

**Fig. 1:**
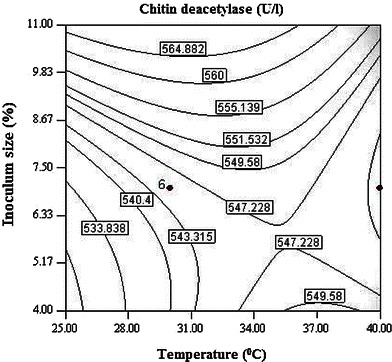
Contour plot of CDA activity as a function of temperature and inoculum size

**Fig. 2:**
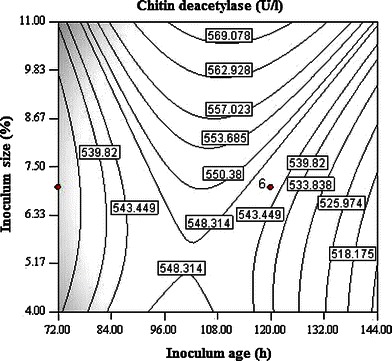
Contour plot of CDA activity as a function of inoculum age and inoculum size

**Fig. 3:**
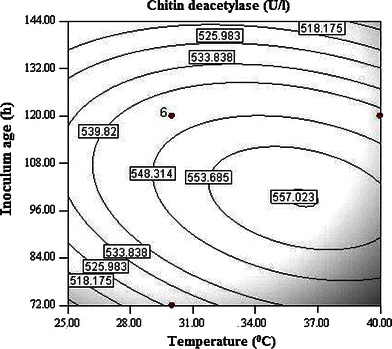
Contour plot of CDA activity as a function of temperature and inoculum age

Interaction between temperature and inoculum size has most significant effect on CDA production (Fig. 1). It is evident from the surface plot that the effect of inoculum size is more pronounced on CDA production as compared to temperature. Higher inoculum size supports CDA production in a positive way while increase in temperature exerted negative effect on production. Inoculum is considered as one of the most important factors affecting enzyme production during fermentation. Higher significance of linear, quadratic and interactive effects of inoculum age and size, suggested their direct relationship with the CDA production. Both the inoculum age and size had a significant quadratic response on enzyme production (Fig. 2). The contour plot demonstrated that high inoculum levels promote CDA production, while using the inoculum of lower age. Decreased CDA production may be observed at high inoculum levels if the optimum for inoculum age shifted toward higher levels. A mutual interactive effect is observed between temperature and inoculum age (Fig. 3), where lower levels of both the factors supported higher enzyme production. pH alone had noteworthy effect on CDA production but its interaction with other independent variables is observed to be non-significant. Although the isolation of fungal strain was not from an environment with extreme pH; however, it had higher levels of CDA production at alkaline pH.

Among the independent variables, all were found to play important role in the CDA production as their *F* values are high and the corresponding *P* values were small (Table 4). The optimum coded values for these parameters were calculated from the model Eq. 2 and were found to be 1.929, −0.416, −1.277, and 1.303 for pH, temperature, inoculum age, and inoculum size, respectively. The corresponding uncoded optimum values are pH, 7.9; temperature, 28.0 °C; inoculum age, 90.0 h and inoculum size, 11.0 % (v/v). Under these conditions, maximum CDA production predicted by the model equation is 648.24 Ul^−1^.

Response surface methodology had been successfully used for enhancement of xylanase production by *Aspergillus carneus* (Fang et al. 2007), endopectinase production by *Aspergillus niger* (Bari et al. 2010), laccase production by *Pleurotus* sp. (Bhattacharya et al. 2011) and protease production by *Brevibacterium linens* (Shabbiri et al. 2012). Statistical optimization of production of various enzymes of chitinolytic enzyme system was attempted by several workers (Vaidya et al. 2003; Gohel et al. 2005; Juarez-Jimenez et al. 2008; Lopes et al. 2008). But most of the studies are related to the optimization of medium components. Only a few reports are available regarding optimization of culture conditions (Nawani and Kapadnis 2005) for chitinase production. To the best of our knowledge, present study is the first attempt to optimize the fermentation conditions for CDA production, hence there is a lack of standard information to compare the current findings. Response surface optimization has also been used for achieving higher product yields (Liu et al. 2008; Jiang 2010).

### Validation of the experimental model

**Fig. 4:**
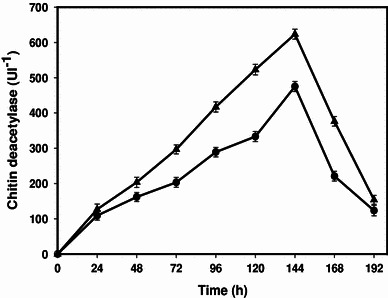
Profile of chitin deacetylase production by mutant *Penicillium oxalicum* SAE_M_-51 under submerged fermentation (*circle* basal conditions; *triangle* optimized conditions)

To validate the model equation, experiments were carried out in triplicate for CDA production under optimum conditions predicted by the model and the CDA production was found to be 623.57 ± 8.2 Ul^−1^ (Fig. 4). The theoretical prediction for the model equation was (648.24 Ul^−1^). The closeness between theoretically predicted values and experimental result at optimum condition validates the model experimentally.

## Conclusions

Statistical optimization of fermentation conditions could overcome the limitations of conventional methods. CCD was proposed to study the combined effects of various culture conditions on CDA production by *P. oxalicum* SAE_M_-51. The existence of interactions between the independent variables with the responses was observed. The experimental CDA production at optimal conditions predicted by the model equation was found to be 623.57 ± 8.2, which is 1.4 times higher than the value obtained (427.08 ± 3.6 Ul^−1^) when parameters kept at their corresponding zero level (Table 1). The optimum fermentation conditions to attain maximum enzyme production are as follows: pH, 7.9; temperature, 28.0 °C; inoculum age, 90.0 h; and 11.0 % inoculum size. Statistical optimization of fermentation conditions under submerged system had led into significant enhancement in CDA production, which further endorse its application for biological production to chitosan.
